# Lower fecal microbiota transplantation ameliorates ulcerative colitis by eliminating oral-derived *Fusobacterium nucleatum* and virulence factor

**DOI:** 10.1186/s13099-024-00633-9

**Published:** 2024-08-08

**Authors:** Dong-Hao Li, Zong-Wei Li, Qi Sun, Lei Wang, Shou-Bin Ning

**Affiliations:** Department of Gastroenterology, Air Force Medical Center of Chinese People’s Liberation Army, Beijing, China

**Keywords:** Ulcerative colitis, Oral pathogenic bacteria, DSS-induced colitis, Fecal microbiota transplantation, 16S rRNA sequencing

## Abstract

**Background:**

Recently, the oral oncobacterium *Fusobacterium nucleatum* (*F. nucleatum*), has been linked with ulcerative colitis (UC). Here, we aim to investigate whether Fecal Microbiota Transplantation (FMT) can alleviate UC by restoring gut microbiota and eliminating oral-derived *F. nucleatum* and virulence factor *fadA*.

**Method:**

C57BL/6J mice were randomly divided into a healthy control group (HC), Dextran Sulfate Sodium group (DSS), oral inoculation group (OR), upper FMT group (UFMT), and lower FMT group (LFMT). Disease activity index, body weight, survival rate, and histopathological scores were used to measure the severity of colitis. The function of the intestinal mucosal barrier was evaluated by performing immunohistochemical staining of the tight junction protein Occludin. Real-time PCR was used to assess the relative abundance of the *nusG* gene and the virulence gene *fadA*. Cytokine levels were detected by ELISA. Full-length sequencing of 16S rRNA was used to analyze the changes and composition of gut microbiota.

**Findings:**

Oral incubation of *F. nucleatum* further exacerbated the severity of colitis and gut dysbiosis. *Peptostreptococcaceae*, *Enterococcaceae*, and *Escherichia coli* were significantly enriched in OR mice. However, LFMT mice showed an obvious decrease in disease activity and were more effective in restoring gut microbiota and eliminating *F. nucleatum* than UFMT mice. *Bacteroidota*, *Lachnospiraceae*, and *Prevotellaceae* were mainly enriched bacteria in LFMT mice. In addition, Genera such as *Lactobacillus*, *Allobaculum*, and *Bacteroidales* were found negative correlation with TNF-α, IL-1β, and IL-6. Genera like *Romboutsia*, *Escherichia Shigella*, *Enterococcus*, and *Clostridium* were found positively correlated with TNF-α, IL-1β, and IL-6.

**Conclusions:**

Oral incubation of *F. nucleatum* further exacerbates the severity and dysbiosis in DSS-induced colitis mice. Besides, lower tract FMT can ameliorate colitis by restoring the gut microbiota diversity and eliminating *F. nucleatum* and virulence factor *fadA*.

## Introduction

Ulcerative colitis (UC) is a chronic, multifactorial, and non-specific inflammatory condition of the colon and rectum, with its main clinical manifestations including diarrhea, hematochezia, abdominal pain, tenesmus, and extraintestinal manifestations [1]. Currently, the etiology of UC is ambiguous. However, research has demonstrated that the occurrence of UC is closely related to the abnormality of the intestinal immune system induced by gut microbiota imbalance. The reduction of symbiotic microbiota and increase of pathogenic bacteria disrupt the intestine’s microecological balance and the disruption ultimately leads to alterations of gut microbiota-related functions, including changes in bacterial fermentation products such as carbohydrates, vitamins, and Short-Chain Fatty Acids (SCFAs), as well as changes in biochemical processes such as immune imbalance [2]. Several comprehensive analyses have revealed significant microbiota differences between healthy individuals and UC patients. The report claimed that the dysbiosis pattern of UC often manifests as a decrease in bacterial diversity and the abundance of symbiotic bacteria such as *Bifidobacteria*, *Bacteroides*, *Eubacteria*, and an increase in *Firmicutes* (especially *Clostridium*) and *Proteobacteria* [3–5]. Unfortunately, until now the specific bacteria that cause UC are not yet elucidated. Evidence from human samples and rodent animal models have demonstrated a high prevalence of specific pathogenic bacteria, such as *Bacteroides fragilis* and *Clostridium difficile*, adherent/invasive *Escherichia coli* (AIEC), *Klebsiella pneumoniae* [6–9], but so far, whether these pathogens are causative factors or only contributing factors in exacerbation of UC is still an ongoing issue.

The “oral-gut microbiota axis” is a concept proposed in recent years to elucidate the complex interactions between oral cavity and intestine microbiota [10]. The oral microbiota, as the second largest microbiota in the human body, plays an important role in oral and systemic health. For healthy individuals, oral and intestinal microbiota are well segregated due to gastric acid, bile acid, and small intestinal rhythmic movements [11]. However, the imbalance of gut microbiota, intestinal mucosal barrier dysfunction, and the use of proton pump inhibitors in UC patients provide feasibility for translocation and ectopic colonization of oral-associated bacteria like *Klebsiella* spp., *Porphyromonas gingivalis*, and *F. nucleatum* to the intestine to participate in the progression of the disease [12]. *Klebsiella* spp. has been identified in healthy human saliva [13]. However, Guo et al. reported that oral *Klebsiella* chaperon usher pili provide site-specific adaptation for the inflamed gut mucosa, demonstrating that oral pathogens are likely to ectopic colonize in the colon [14]. On the other hand, oral manifestations can serve as potential signs of UC. Reports suggested that oral lesions, particularly recurrent aphthous ulcers and periodontitis, can be utilized as mucocutaneous signs to determine the occurrence and severity of UC, which would facilitate the early diagnosis of UC [15]. These studies showed that periodontitis and UC are both caused by the same microorganisms and immune pathogenesis, as they constitute an ‘oral-intestinal’ axis and interact with each other, resulting in a vicious cycle.

*F. nucleatum* is an opportunistic pathogen that resides in the oral cavity and plays a core role in the formation of oral plaque biofilm. It is reported that the positive rates of *F. nucleatum* and *fadA* in patients with periodontal diseases such as gingivitis and periodontitis are significantly increased [16]. However, *F. nucleatum* is more than an oral pathogen. Current evidence tends to indicate that *F. nucleatum* is a contributing factor for gastrointestinal diseases such as colorectal cancer and UC [17–19]. Mara R. et al. proposed a “two-hit” model in colorectal carcinogenesis, with *F. nucleatum* as the second hit exacerbating cancer progression and identified *F. nucleatum* as cancer “facilitators“ [17]. Lin S et al. revealed that *F. nucleatum* may exacerbate UC by promoting intestinal microbiota imbalance and metabolic disorders [18]. Su W et al. suggested that *F. nucleatum* may contribute to UC by activating autophagic cell death [19]. In this context, it is valuable to study the role of oral pathogen *F. nucleatum* in promoting disease and gut dysbiosis in UC.

Fecal microbiota transplantation (FMT) is a biotherapy of transferring preparative fecal homogenate from healthy donors into the gastrointestinal tract of patients via colonoscopy or enema, to restore patients’ intestinal flora and to achieve therapeutic benefits [20]. Multiple clinical evidence has demonstrated that FMT reduces disease activity and obtains remission in UC patients [21, 22]. However, the specific bacteria and effective ingredients that play key roles of FMT in the treatment of UC are still largely unknown. Hourigan et al. found that FMT treatment can effectively eliminate *Clostridium* difficile (*C. difficile*) from the intestines of *C. difficile* infection (CDI) patients and enhance bacterial diversity in Inflammatory Bowel Disease (IBD) patients and no *cdtB* virulence gene of *C. difficile* was detected in their stool samples at 3 and 6 months after FMT [23]. Julia et al. detected pro-carcinogenic bacteria *Bacteroides fragilis*, *F. nucleatum*, and *Escherichia coli* virulence genes (*bft*, *fadA*, *pks*) prior and post FMT in recurrent CDI patients, revealing that those pro-carcinogenic bacteria virulence genes to have reduced levels or were not detected post-FMT [24]. These studies indicated that FMT plays a therapeutic role by enriching specific bacteria and reducing pathogenic bacteria and virulence factors, which provides therapeutic strategies for the treatment of UC.

In our previous work, we found oral *F. nucleatum* disseminated to the colon and secreted virulence FadA adhesin to bind the E-cadherin protein located in the mucosal epithelium, further activating the NF-κ signaling pathway and facilitating colitis. The present study aims to evaluate the alterations of gut microbiota in DSS-induced colitis mice and investigate how oral *F. nucleatum* contributes to dysbiosis, and whether FMT can alleviate colitis by eliminating *F. nucleatum* and its virulence factor in the gut.

## Materials and methods

### Bacterial culture

*F. nucleatum* strain ATCC 25586 was used in this study. ATCC 25586 were inoculated on blood agar medium and anaerobic cultured for 2 days at 37℃, then a single colony was transferred to 10 ml BHI medium containing vitamin K (0.2 µg/ml) and hemin chloride (5 µg/ml) for 2 days. As previously described, the substrate containing ATCC25586 then centrifugation at 4000r/min for 10 min, washed with PBS, and centrifugated again for 10 min and diluted with PBS to 1 × 10^9^ CFU/ml.

### Construction of colitis mice

Male C57BL/6J Specific Pathogen Free (SPF) mice aged 6–8 weeks were used in this study. The feeding and experimental procedures were conducted strictly in compliance with ARRIVE guidelines (ARRIVE 2.0) and international laws and policies (Guide for the Care and Use of Laboratory Animals). To ensure the consistency of microbiota and promote colonization, streptomycin (2 mg/ml) was added to drinking water for 3 consecutive days before the experiment was conducted. To induce colitis, 2.5% (w/v) dextran sulfate sodium (DSS) solution was prepared by dissolving in mice drinking water for 14 days.

Thirty C57BL/6J SPF mice were randomly divided into the Healthy control group (HC), Dextran Sulfate Sodium group (DSS), Oral inoculation group (OR), and Upper FMT group (UFMT), Lower FMT group (LFMT), with 6 mice in each group. Mice in the HC group were given normal drinking water and diet, DSS mice were given 2.5% DSS solution for 14 days, and OR mice were subjected to oral inoculation with PBS suspension containing ATCC25586 (concentration: 1 × 10^9^CFU/ml) daily for 2 weeks besides DSS solution intake. UFMT mice were given fecal suspension originating from healthy mice donors by oral gavage in addition to the DSS intake and oral inoculation. LFMT mice were given donor fecal suspension by enema daily in addition to DSS intake and oral inoculation.

Mice body weight, stool formation, hematochezia, and mortality of all mice were daily recorded. Fecal samples were collected every day and stored at -80℃. Mice were sacrificed and the samples were collected for further detection after 14 days of interventions.

### Implementation of FMT

Fresh feces were collected from four healthy mice. The collected feces were then mixed with sterile normal saline in a container with a concentration of 0.125 g/ml and homogenized immediately. Centrifuge the homogenate at 1200/rpm for 5 min and collect fecal sediment for transplantation. A suitable-sized needle was used for gastric gavage or enema. The amount of gastric gavage or enema is 0.2 ml/10 g per mouse, carefully taken to avoid regurgitation.

### Measurement of colitis severity

The disease activity of mice was evaluated by body weight loss, Stool formability, and degree of hematochezia. Disease activity index **(**DAI) was calculated as combined scores of weight loss, stool formability, and hematochezia ranging from 0 to 12. Hematoxylin and eosin (HE) staining was performed to calculate the histopathological score. The histological score is calculated as follows: damage range: 0: n/a, 1: ≤ 25%, 2: ≤ 50%, 3: ≤ 75%, 4: ≤ 100%. Gland mucosal loss: 0 = none, 1 = mild, 2 and 3 = moderate, 4 = severe. Tissue damage: 0 = no mucosal damage, 1 = scattered epithelial lesions, 2 = surface mucosal erosion or focal ulcer, 3 = extensive mucosal damage extending to deeper structures. Inflammatory cell infiltration: 0 = occasional inflammatory cells in the lamina propria, 1 = increased inflammatory cells in the lamina propria, 2 = confluence of inflammatory cells and extension to the submucosa, 3 = infiltration, and transmural extension. The total score is determined by the sum scores of each category.

### Immunohistochemical staining

Mice colon specimens were dehydrated and embedded in wax and cut into 4-µm tissue slices using a microtome. After dewaxed and hydrated, the tissue sections were placed on a high-temperature resistant section rack in a beaker, and an appropriate amount of repair solution (0.01M EDTA buffer, pH 9.0) was added for antigen repair. Prepared 3% hydrogen peroxide was added to block endogenous peroxidase, then incubated at room temperature for 15 min, and washed three times with PBS. By diluted normal goat serum was added and blocked for 30 min at room temperature. Then add primary antibodies and incubate overnight at 4 °C. HRP-labeled goat anti-rabbit/mouse secondary antibody was added and incubated at 37 °C for 30 min. DAB color solution was added and then counterstained with Mayer hematoxylin for 2 min. After sealing the slides, the images were observed under a microscope.

### Realtime-PCR

Colon tissue specimens were thoroughly ground in a mortar filled with liquid nitrogen, and total bacterial DNA was extracted using a DNA extraction kit. The procedures were strictly according to the manufacturer’s instructions. Nano-Drop 2000 was used to determine the concentration of extracted DNA. The real-time amplification system was performed by automatic thermocycler, with 10µL SYBR Green Master, 1µL upstream and downstream primers, 6µL ultrapure water, and 2µL DNA template. The reaction conditions were as follows: 95℃, 10 min; 95℃, 15 s, 60℃, 15 s; 72℃, 30s and 72℃ for 40 cycles. The primer set for *F. nucleatum* was designed to target the specific conserved *nusG* gene. Premier 5.0 software was used to design the primers. *nusG*: Forward: 5’-GTT AGA GGA AAG CCC AAG AAG GTC-3’; Reverse: 5’-AGG AAT AGG GTC AGA ACC AAC TCC-3’; *fadA*: Forward: 5’-CAC AAG CTG ACG CTG CTA GA-3’, Reverse: 5’-TTA CCA GCT CTT AAA GCT TG-3’.

### 16 S rRNA full-length sequencing

Fecal samples were collected and stored at -80℃. Genomic DNA from samples was extracted using the QIAamp Fast DNA Stool Mini Kit. The concentration and purity of extracted DNA were analyzed by agarose gel electrophoresis. Primers (27 F: AGR GTT TGA TYN TGG CTC AG and 1492 R: TAS GGH TAC CTT GTT ASG ACT T) were used to amplify the full-length 16 S rRNA gene. Purified PCR products were used to construct sequencing libraries using the PacBio platform (Biomarker-technologies Company, Beijing) according to the manufacturer’s protocol. The constructed libraries were subjected to library quality inspection, and the qualified libraries were sequenced by PacBio Sequel. The obtained high-quality sequences were clustered into operational taxonomic units (OTUs) with 97% similarity. α-diversity, β-diversity, and Metastats analyses were performed using BMK Cloud (www.biocloud.net).

### Enzyme-linked immunosorbent assay

The cytokine levels in colon tissue were measured using Enzyme-linked immunosorbent assay (ELISA). To extract colon proteins, colon tissues were sliced into small pieces and ground in a homogenizer, after mixing with 400 µl tissue lysate, the mixture was homogenated with ultrasonication. Then transferred the lysis solution to a 1.5 ml centrifuge tube, and centrifuged at 12,000/rpm for 5 min at 4℃, the supernatant was collected and protein concentration was measured following the manufacturer’s kit.

### Statistical analysis

The categorical variables were analyzed using Pearson’s Chi-square test and Fisher’s exact test. Continuous data conforming to the normal distribution and homogeneity of variance were analyzed using One-way ANOVA. The measurement data that did not meet the normal distribution were described as non-parametric tests (Kruskal-Wallis). All statistical procedures were conducted via IBM SPSS Statistics 21.0. The value of *p* < 0.05 was considered statistically significant.

## Results

### *F. nucleatum* further aggravated inflammation and gut barrier damage in DSS-induced colitis mice

**Fig. 1:**
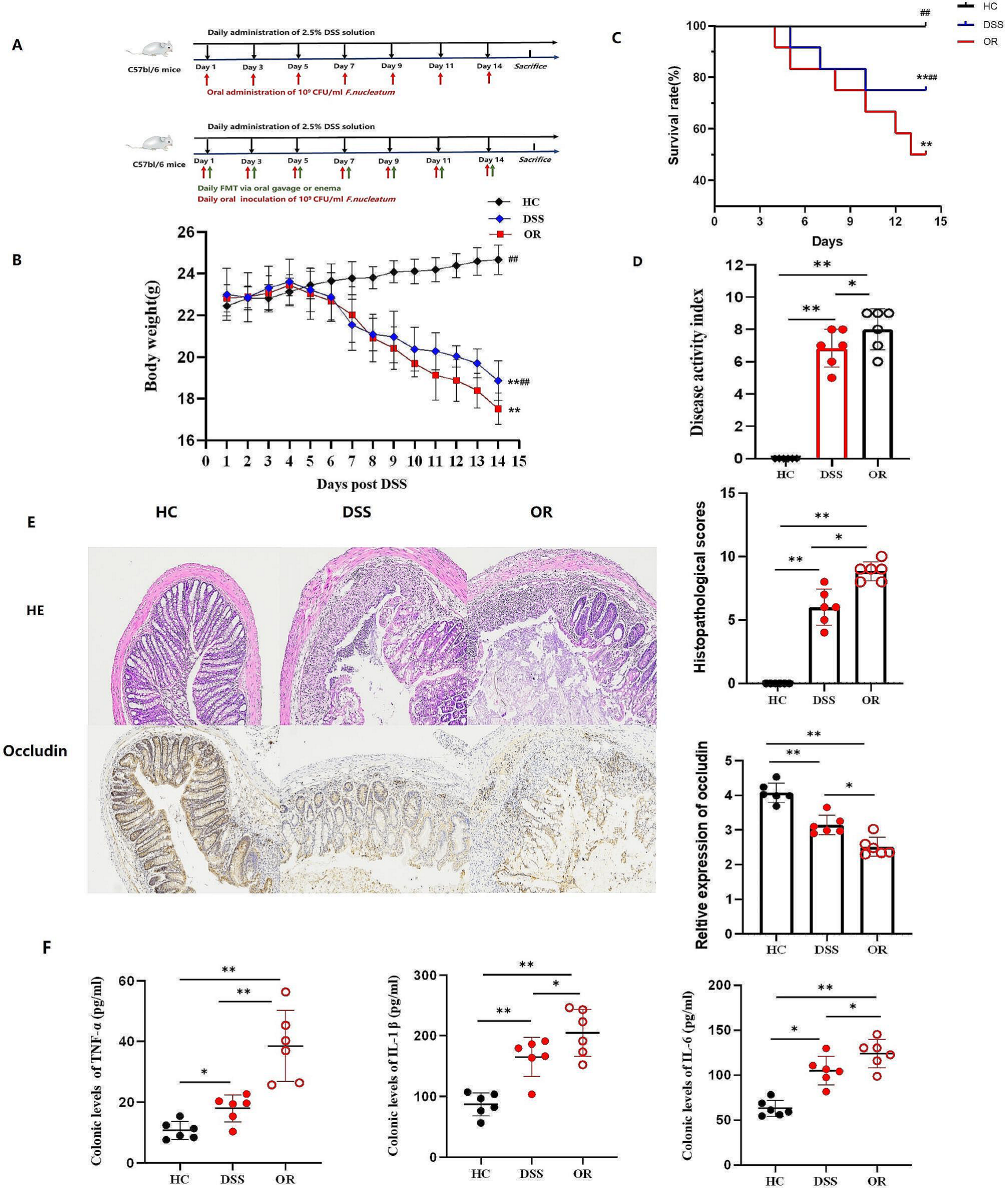
Oral-infected *F. nucleatum* further aggravated inflammation and gut barrier damage in DSS-induced colitis mice. **A,** A flowchart of animal experiments in this study. **B,** Variations of mice body weight among HC, DSS, and OR groups. **C,** Survival rate of the three groups (%). **D,** Disease activity index scores of the three groups. **E,** Representative images of histopathological by HE and immunohistochemical staining for Occludin protein. The histopathological scores and relative expression of Occludin protein were also calculated. **F,** Colonic levels of TNF-α, IL-1β and IL-6 by ELISA. **B,** Data are presented as mean ± S.D. *p* values were determined by analysis of variance, **p<0.01 vs. HC, ^##^*p*<0.01 vs. OR. C, Data were analyzed by Pearson’s Chi-square test. **p<0.01 vs. HC, ^##^*p*<0.01 vs. OR. D-F, Data are presented as mean ± S.D. *p* values were determined by analysis of variance, *p<0.05, **p<0.01

To investigate whether *F. nucleatum* promotes inflammation and gut barrier damage in DSS-induced colitis mice, ATCC25586 strains were orally administrated daily to SPF C57BL/6J mice besides 2.5% DSS solution intake. The animal experiment procedure is shown in Fig. 1A. Body weight, survival rate, and disease activity index were monitored among groups. In HC mice, the body weight increased gradually over time, DSS and OR mice witnessed a slight increase at the first four days, and decreased from the fifth day. The body weight of OR mice decreased obviously in contrast with HC and DSS mice at the end of the experiment (*p*<0.01, Fig. 1B). All mice survived in the HC group, which was significantly higher than DSS and OR mice. In addition, DSS mice manifested a higher survival rate than OR mice (*p*<0.01, Fig. 1C). The disease activity index of OR mice was significantly higher than HC and DSS mice (*p*<0.05, Fig. 1D). HE staining showed the intestinal structure of HC mice was continuous and intact, with no inflammatory cell infiltration. Disrupted intestinal structure and inflammatory cell infiltration were observed in DSS mice. In the OR group, extensive mucosal damage and severe gland mucosal loss, with the confluence of inflammatory cell infiltration were observed. Histopathological scores were calculated as shown in Fig. 1E. Immunohistochemical staining revealed that Occludin protein was continuously expressed in the epithelium in HC mice, but the expression was interrupted in DSS and OR mice. In addition, the expression in DSS mice was significantly higher than in OR mice (*p*<0.05, Fig. 1E). We also observed increased levels of inflammatory cytokines TNF-α, IL-1β, and IL-6 in two colitis groups (Fig. 1F). Moreover, the cytokines levels were remarkably increased in OR mice in contrast with DSS mice (*p*<0.05, Fig. 1F).

### *F. nucleatum* promotes dysbiosis in DSS-induced colitis mice

**Fig. 2:**
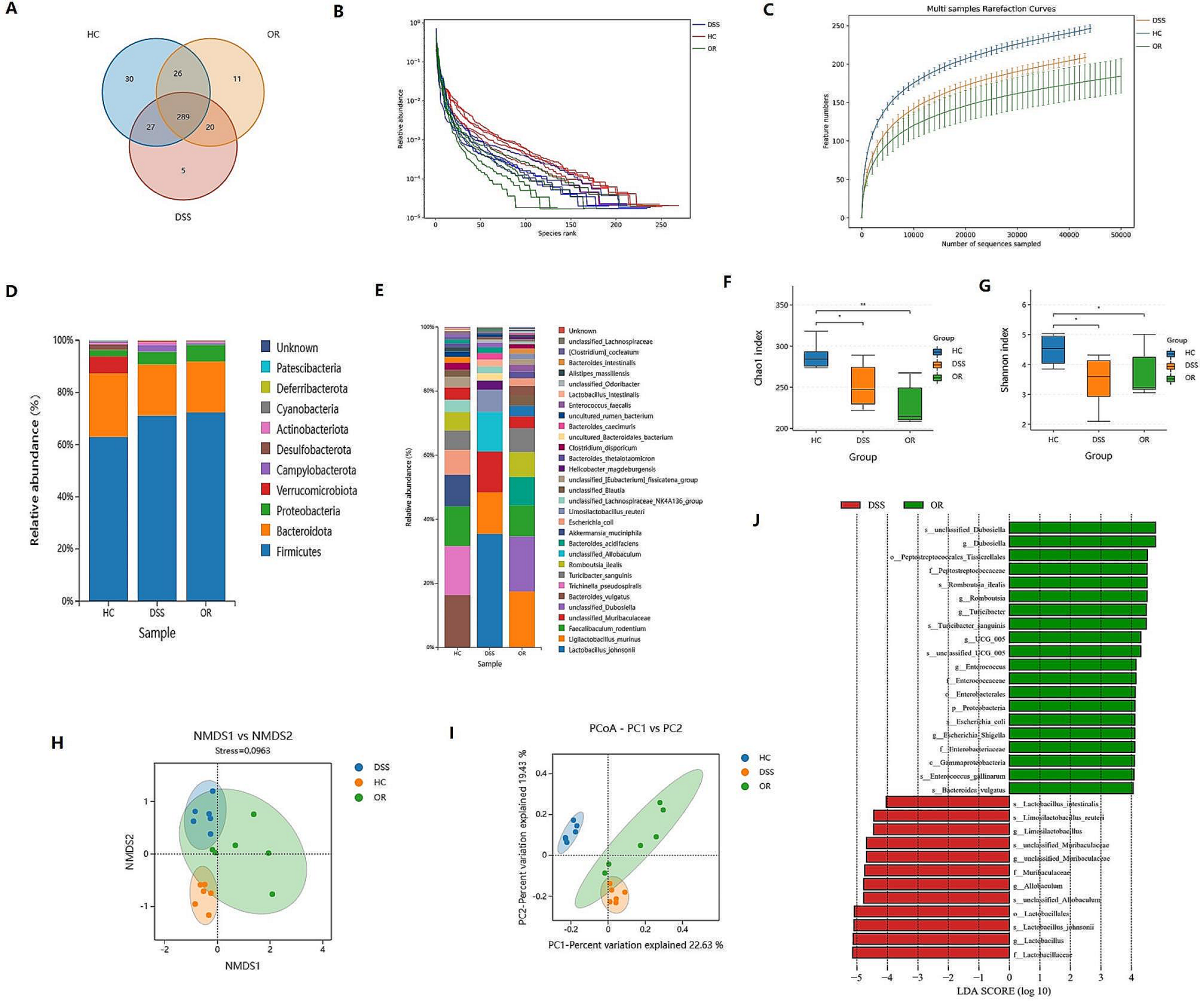
*F. nucleatum* further aggravates the dysbiosis in DSS-induced colitis mice. A, The Venn diagram analysis of OTUs among HC, DSS, and OR groups. B, Rank-abundance curves reflect higher species richness was found in HC mice(red curve) than in DSS and OR mice. The species richness of DSS mice was more abundant than OR mice. C, Rarefaction curves among the three groups. D, E, Relative abundance of microbiota composition on phylum and species levels. F, G, Alpha diversity including Taxa richness (Chao1 index) and species diversity (Shannon index) reflect that the microbial richness and diversity of HC mice were higher than DSS and OR mice. H, Non-metric multidimensional scaling, NMDS analysis reflects the similarity in microbial composition of samples. I, Principal coordinates analysis (PCoA) reflected the differences in sample species diversity among groups. J, Histogram represents the enriched microbiota in DSS and OR groups from phylum level to species level. F-G, Data are presented as mean ± S.D. *p* values were determined by analysis of variance, *p<0.05, **p<0.01

Next, we sought to investigate the effect of *F. nucleatum* on disturbing the microbiota in DSS mice. We performed oral incubation of ATCC25585 strains to C57BL/6J mice. The Venn diagram analysis of OTUs revealed that 408 OTUs were identified in the three groups. HC, DSS, and OR groups contained 30, 5, and 11 unique OTUs, respectively (Fig. 2A). The species rank curve revealed a smoother and wider curve in the HC group, and the sharpest decline was found in the OR group (Fig. 2B). The rarefaction curve indicated the sequencing quality was in according with the requirement for further analysis (Fig. 2C). We further analyzed the fecal microbiota compositions at phylum and genus levels (Fig. 2D and E). The dominant bacterium in HC mice were *Firmicutes*, *Bacteroidota*, and *Verrucomicrobiota*. However, an increased abundance of *Firmicutes* and *Proteobacteria* was found in DSS and OR mice. The dominant bacterium in DSS and OR mice were *Firmicutes*, *Bacteroidota*, and *Proteobacteria.* At the same time, alpha and beta diversity were utilized to evaluate the differences in microbiota composition and diversity. The alpha diversity including the Shannon index and Chao1 index was remarkably reduced in DSS and OR mice in contrast with HC mice, and OR mice were significantly lower than DSS mice (*p*<0.05, Fig. 2F and G). The beta diversity including Non-Metric Multidimensional Scaling (NMDS) and Principal Coordinates Analysis (PCoA) reflected the intestinal microbiota distributions were cluster separated among groups (Fig. 2H and I). Histogram from the phylum level to the genus level revealed the significantly enriched bacterium in OR mice were mainly *Dubosiella*, *Peptostreptococcaceae*, *Romboutsia*, *Enterococcaceae*, and *Escherichia coli*. DSS mice enriched bacterium mainly include *Lactobacillaceae*, *Allobaculum*, and *Muribaculaceae* (Fig. 2J).

### LFMT alleviates inflammation and gut barrier damage in DSS-induced colitis mice

**Fig. 3:**
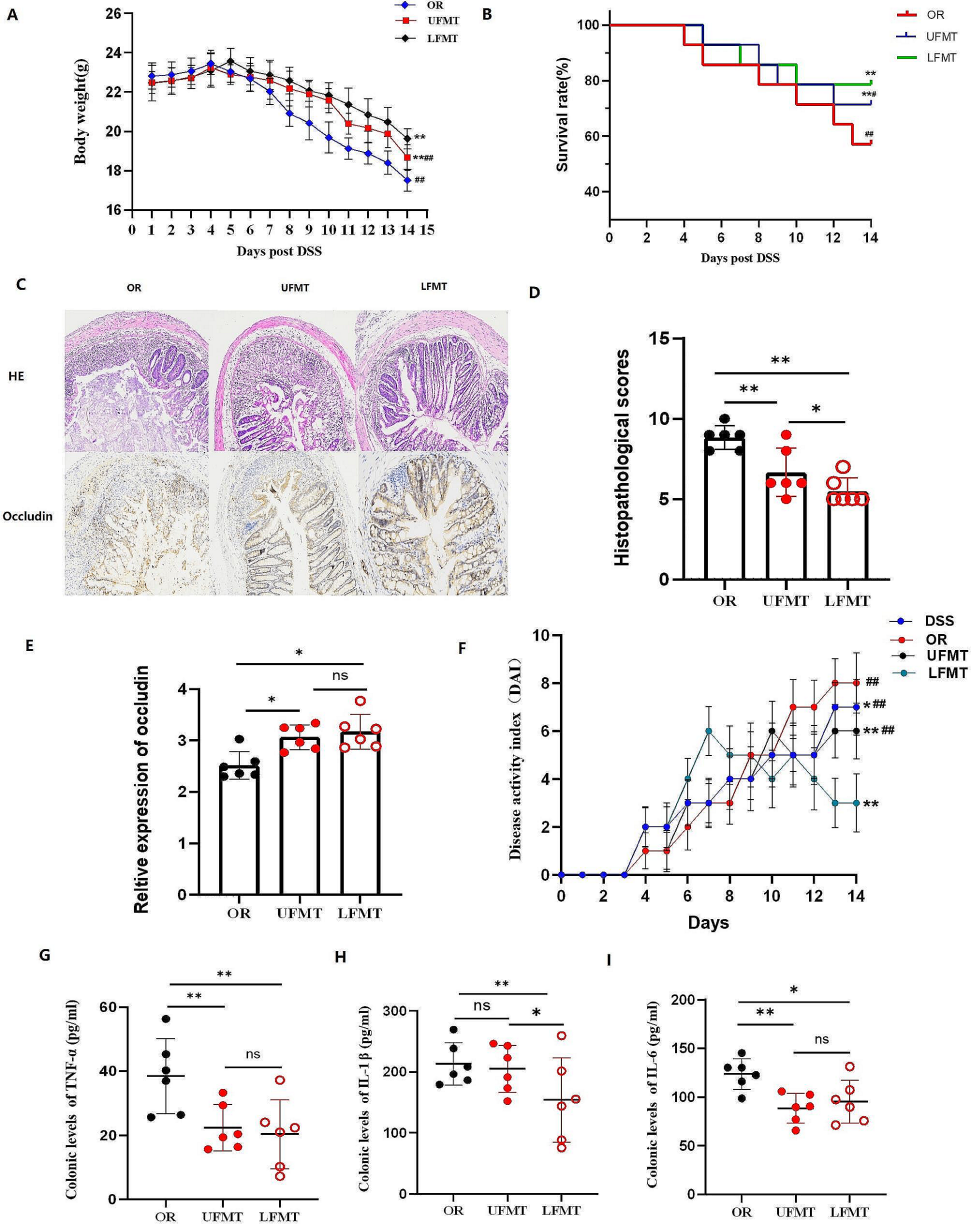
Fecal microbiota transplantation via the lower gastrointestinal tract alleviates inflammation and gut barrier damage in DSS-induced colitis mice. **A,** Body weight changes among OR, UFMT, and LFMT groups. **B,** Survival rate (%). **C,** Representative images of histopathological by HE and immunohistochemical staining for Occludin protein. **D, E,** Histopathological scores and relative expression of Occludin protein. **F,** Variations of disease activity index among DSS, OR, UFMT, and LFMT groups. **G, H, I,** Colonic levels of TNF-α, IL-1β, and IL-6 by ELISA. **A,** Data are presented as mean ± S.D. *p* values were determined by analysis of variance, **p<0.01 vs. LFMT, ^##^*p*<0.01 vs. OR. **B,** Data are performed by Pearson’s Chi-square test. **p<0.01 vs. OR, ^##^*p*<0.01 vs. LFMT. **D, F,** Data are performed by Kruskal-Wallis test. **p<0.01 vs. OR, ^##^*p*<0.01 vs. LFMT. **E, G-I,** Data are presented as mean ± S.D. *p* values were determined by analysis of variance, *p<0.05, **p<0.01

Since we demonstrated *F. nucleatum* further facilitates inflammation and gut structure damage in DSS mice, we next investigated the effect of upper FMT and lower FMT on inflammation and intestinal barrier dysfunction. The body weight of UFMT and LFMT dropped not as sharply as OR mice (Fig. 3A). Survival curves declined markedly in OR and UFMT mice, and LFMT mice survival rate remained flat curves (Fig. 3B). The histopathological changes by HE staining reflected severe mucosal damage and a large number of inflammatory cells infiltrated in OR mice. However, we observed remained integrity of intestinal epithelium and scattered inflammatory cell infiltration in LFMT mice. Although ameliorative manifestations were observed in the intestine of UFMT mice, the intestinal structure was still damaged and remained few gland structures. The histopathological scores supported the results (Fig. 3C and D). We also observed increased Occludin protein expression in the LFMT mice, which was significantly different from the OR and UFMT groups (*p*<0.05, Fig. 3C and E). The disease activity of LFMT mice continued to increase in the first week and then decreased significantly from the first week. At the end of the experiment, the disease activity of LFMT mice was significantly different from DSS, OR, and UFMT groups (*p*<0.01, Fig. 3F). The disease activity index of UFMT mice remained unchanged from the tenth day. The OR mice had the highest disease activity at the end of the experiment, which was significantly different from other groups (*p*<0.01, Fig. 3F). We observed a remarkable reduction of TNF-α and IL-6 levels in UFMT and LFMT mice (*p*<0.05, Fig. 3G and I). Compared with OR and UFMT, the level of IL1-β was significantly downregulated in LFMT mice (*p*<0.05, Fig. 3H).

### LFMT restores intestinal microbiota dysbiosis in DSS-induced colitis mice

**Fig. 4:**
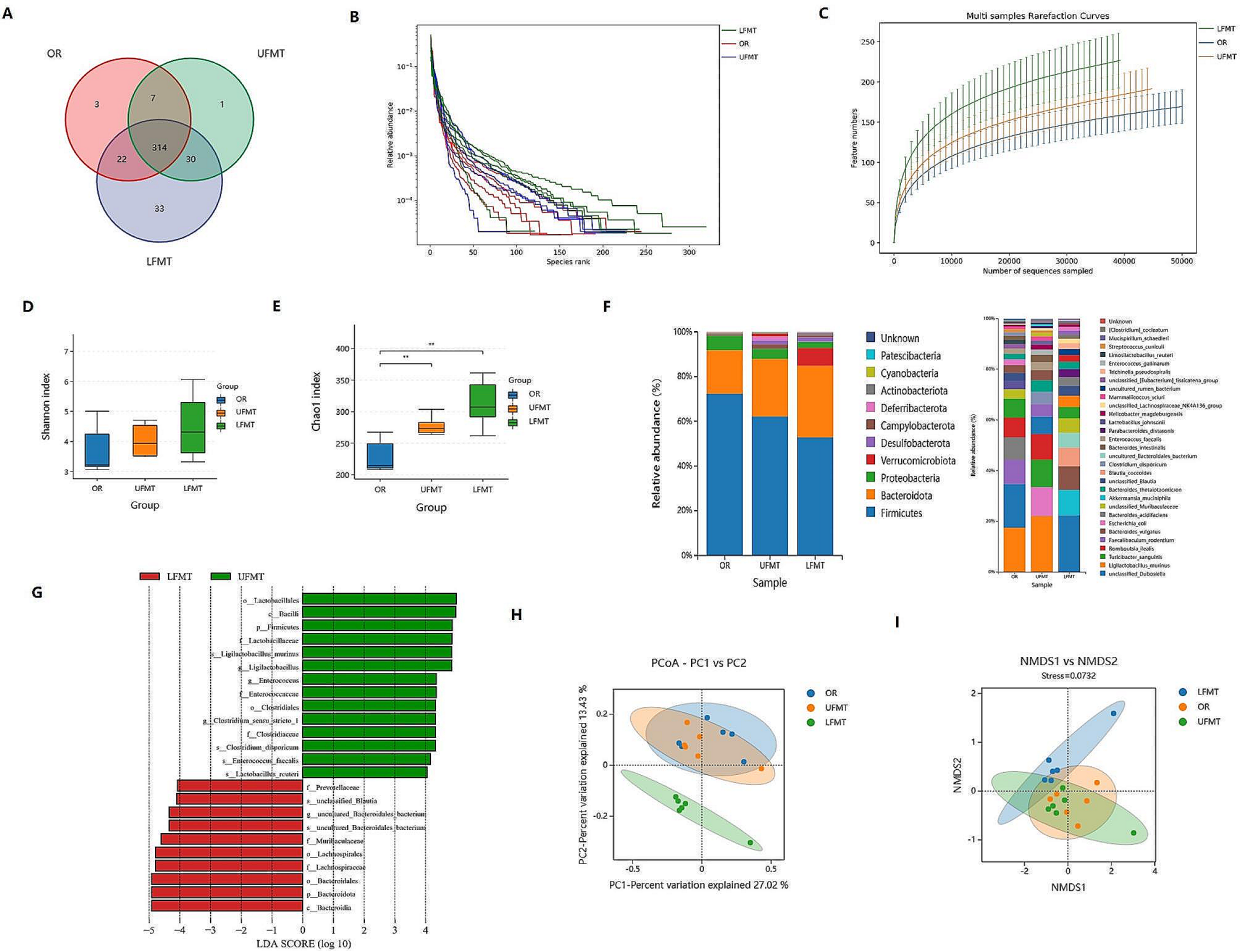
Fecal microbiota transplantation from the lower gastrointestinal tract restores intestinal microbiota dysbiosis in DSS-induced colitis mice. **A,** The Venn diagram analysis of OTUs among OR, UFMT, and LFMT groups. **B,** Rank-abundance curves reflect higher species richness in LFMT mice (green curve) than in OR (red curve) and UFMT (blue curve) mice. **C,** Rarefaction curves among the three groups. **D, E,** Taxa richness (Chao1 index), and species diversity (Shannon index) reflect that the microbial richness and diversity of LFMT mice were higher than OR and UFMT mice. **F,** Relative abundance of microbiota composition on phylum and species levels. **G,** Histogram represents the enriched microbiota in UFMT and LFMT groups from phylum level to species level. **H,** NMDS analysis reflects the similarity in the microbial composition of samples. **I**, PCoA analysis reflected the differences in sample species diversity among groups. **D-E**, Data are presented as mean ± S.D. *p* values were determined by analysis of variance, *p<0.05, **p<0.01

To investigate the effect of FMT on intestinal microbiota dysbiosis in DSS-induced colitis mice, we performed upper and lower FMT in C57BL/6 mice and collected feces samples for 16 S-rRNA full-length sequencing. The Venn diagram of OTUs revealed 410 OTUs were identified in the OR, UFMT, and LFMT mice. Besides, OR, UFMT, and LFMT groups contain 3, 1, and 33 unique OTUs, respectively (Fig. 4A). The species rank curve revealed a smoother and wider curve was observed in the LFMT group, and the sharpest declines were found in the OR group (Fig. 4B). The rarefaction curve is shown in Fig. 4C. The species diversity revealed an increased Shannon index in the LFMT group, but no significant difference compared with OR and UFMT groups (*p*>0.05, Fig. 4D). Taxa richness (Chao1 index) was remarkably increased in UFMT and LFMT, and OR mice were significantly lower than DSS mice (*p*<0.01, Fig. 4E). We further analyzed the microbial compositions of the feces samples at phylum and genus levels. As was shown in Fig. 4F, an increased abundance of *Bacteroidota* and *Verrucomicrobiota* was found in UFMT and LFMT mice. We also observed a decreased abundance of *Firmicutes* and *Proteobacteria* in UFMT and LFMT mice. In addition, a histogram from the phylum level to the genus level revealed the significantly enriched bacterium in UFMT were *Lactobacillales*, *Enterococcaceae*, and *Clostridium*. LFMT mice enriched bacterium mainly include *Bacteroidota*, *Lachnospiraceae*, and *Prevotellaceae* (Fig. 4G). NMDS and PCoA analysis indicated the intestinal microbiota distributions of LFMT were cluster-separated from DSS and UFMT groups (Fig. 4H and I).

### *F. nucleatum* and virulence *fadA* levels are negatively correlated with LFMT therapeutic times

**Fig. 5:**
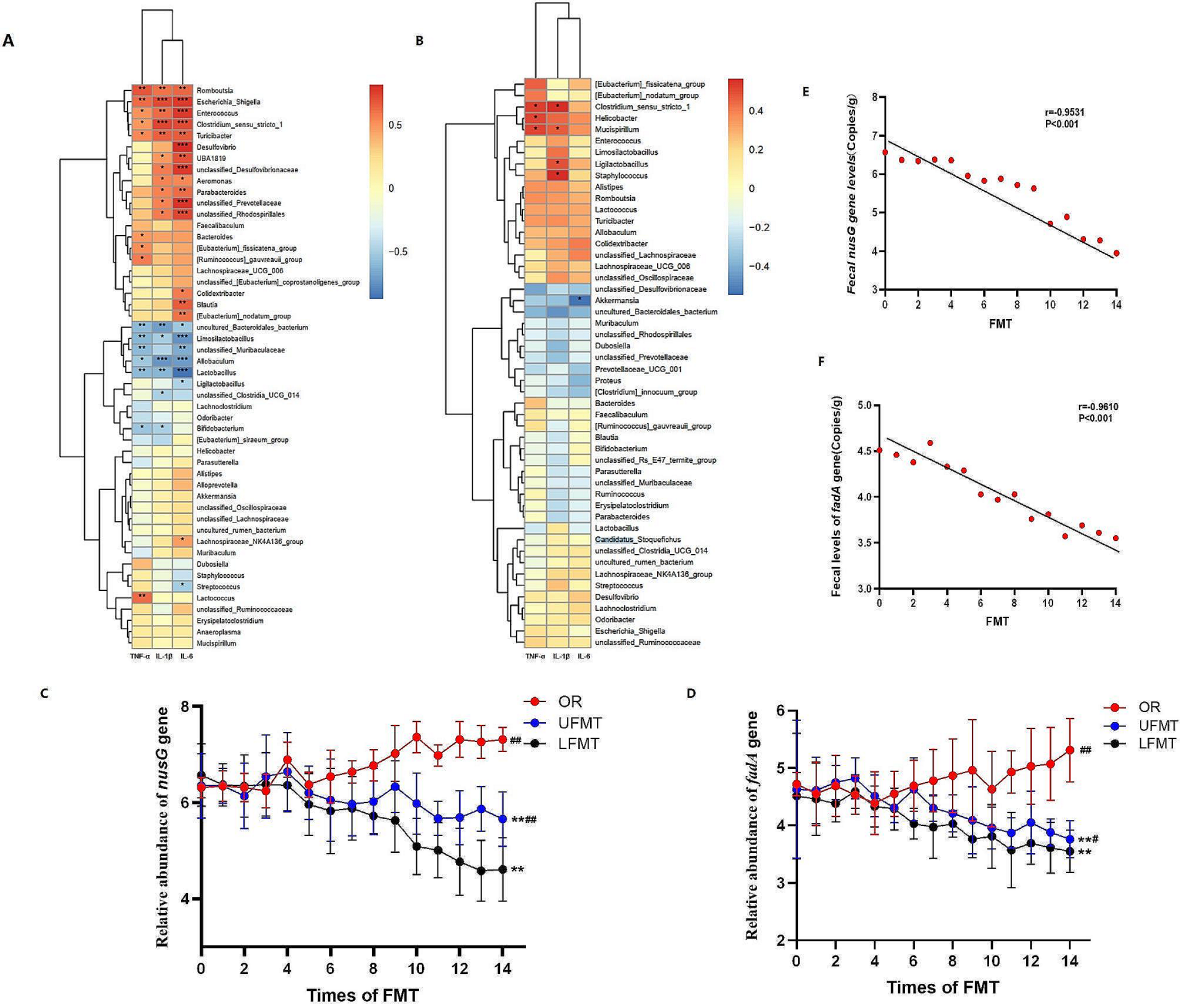
Lower gastrointestinal FMT can reduce the levels of *F. nucleatum* (shown as *nusG* gene) and virulence *fadA*, and the levels of *F. nucleatum* and virulence *fadA* are negatively correlated with FMT therapeutic times. **A,** The Correlation heatmap reveals the correlation between inflammatory cytokines TNF-α, IL-1β, IL-6, and the top 50 genera in abundance among the five groups. **B,** Correlation heatmap among OR, UFMT, and LFMT groups. **C,** The changes of *nusG* gene level with the increased times of FMT among groups. **D,** The changes of *fadA* gene level with the increased times of FMT among groups. **E,** Fecal *nusG* level was negatively correlated with FMT times (*r* = 0.9531, *p*<0.001). **F,** Fecal *fadA* level was negatively correlated with FMT times (*r* = 0.9610, *p*<0.001). **C, D**, Data are presented as mean ± SD, *p* values were determined by analysis of variance, *p<0.05, **p<0.01 vs. OR, ^##^*p*<0.01 vs. LFMT. **E, F**, and Data are performed by Spearman correlation

The correlation heat map illustrated the relationship between the inflammatory factors TNF-α, IL-1β, and IL-6 and the abundance of the top 50 genera among five groups and between UFMT and LFMT groups. Genera usually deficient probiotics in IBD such as *Lactobacillus*, *Allobaculum*, and *Bacteroidales* were found negative correlation with TNF-α, IL-1β, and IL-6. Genera like *Romboutsia*, *Escherichia Shigella*, *Enterococcus*, and *Clostridium* were found positively correlated with TNF-α, IL-1β, and IL-6 (Fig. 5A and B). We also analyzed *nusG* and *fadA* levels at different time points using real-time PCR. We observed that *nusG* and *fadA* were continuously increased in OR mice, which was significantly higher than UFMT and LFMT (*p*<0.01, Fig. 5C and D). Remarkable reduction was found in UFMT and LFMT, but the declined trend of UFMT is not as obvious as in LFMT mice (*p*<0.05, Fig. 5C and D). In addition, fecal *nusG* and *fadA* gene levels were negatively correlated with LFMT therapeutic times (*r* = 0.9531, *p*<0.001, Fig. 5E) (*r* = 0.9610, *p*<0.001, Fig. 5F).

## Discussion

*F. nucleatum* is considered as an opportunistic pathogen that usually resides in the oral cavity and gastrointestinal tract. The oral pathogens are implicated in many diseases such as periodontitis, gingivitis, oral cancer, inflammatory bowel disease, and colorectal cancer [25–27]. In previous reports, *F. nucleatum* was found enriched in tumor tissues of breast cancer patients [28], and there was also a report that *F. nucleatum* found in umbilical cord blood of premature infants is associated with adverse birth events such as stillbirth and termination of labor [29]. These studies suggested that *F. nucleatum* is more than an oral pathogen. However, adherence to host cells is a prerequisite for the pathogenicity of *F. nucleatum*. As an adhesive microorganism, *F. nucleatum* can adhere to the surface of a variety of host cells, such as epithelial cells, fibroblasts, endothelial cells, monocytes/macrophages, and bind to salivary macromolecules, extracellular matrix proteins, antibodies, and trigger a series of host immune responses. The key virulence FadA adhesin, expressed on the surface of *F. nucleatum*, serves as a scaffold for biofilm formation and improves the acid resistance and external resistance of *F. nucleatum* [30]. Rubinstein et al. reported that FadA mediates attachment and invasion and promotes colorectal carcinogenesis by binding to E-cadherin proteins on colorectal cancer cells [31]. Hong et al. reported that *F. nucleatum* outer membrane vesicles containing the virulence determinant FadA translocate into the joints, triggering local inflammatory responses [32]. Those studies unveiled that FadA is a crucial pathogenic factor for *F. nucleatum*.

UC is a chronic inflammatory disease driven by a complex interplay between a dysregulated immune microbiota and the host immune system.

The destruction of the epithelial barrier and gut microbiota dysbiosis provide a prerequisite for the colonization and invasion of oral pathogens, when these pathogens translocate to the intestine, they become pro-inflammatory factors in the gut. Several studies have demonstrated that *F. nucleatum* was found to accumulate in UC patients and to be associated with clinical features [33, 34]. Li et al. reported that the fecal *F. nucleatum* harbored virulence *fadA* gene was increased in UC patients compared with the control group, and was related to the severity and location of UC [33]. Chen et al. reported that *F. nucleatum* was enriched in 51.78% of UC tissues and correlated with the clinical course, clinical activity, and refractory behavior of UC [34]. In this study, we first investigated whether oral infection of *F. nucleatum* could aggravate UC inflammation in a DSS-induced colitis animal model. Our results demonstrated that oral inoculation of *F. nucleatum* further aggravates disease activity and epithelial barrier disruption in DSS-induced mice.

The gut microbiota of UC patients is different from healthy individuals. Compared with healthy people, the microbial profiles of IBD patients manifested as a reduction of symbiotic bacteria and an increase of opportunistic pathogens. Hirano et al. found the diversity of microbiota was reduced in both inflamed and non-inflamed colon tissues of IBD patients [35]. El-Baz et al. witnessed a significant increase in *Escherichia coli* and *Fusobacterium* and a decrease in *Bifidobacteria* in UC patients [36]. Lin S et al. confirmed that *F. nucleatum* could further aggravate inflammation and barrier damage in a colitis model induced by DSS solution, reduce the level of *Bifidobacterium* and *Faecalibacterium*, and increase the abundance of *Escherichia coli* and *Shigella* [18]. In this experiment, DSS mice manifested significant microbiota dysbiosis, with an increased abundance of *Firmicutes*, and *Proteobacteria* and a decreased abundance of *Bacteroidetes*, and *Verruobacteria*. *F. nucleatum* further promoted the gut dysbiosis of the microbial community. The α-diversity and β-diversity of microbiota in OR mice remarkably reduced to DSS mice. Besides, some pathogenic bacteria such as *Dubosiella*, *Peptostreptococcaceae*, *Romboutsia*, *Enterococcaceae*, and *Escherichia coli* gathered in the feces of OR mice.

In recent years, FMT has been a novel therapy that obtained therapeutic benefits by preparing selected donor feces into lyophilized substances in vitro and perfusing them into the intestinal tract of patients. FMT was initially used in the treatment of refractory CDI patients with notable curative effectiveness [37]. Therefore, FMT becomes a promising therapeutic method for the treatment of FMT. However, the long-term efficacy and adverse effects of FMT have not been fully elucidated. The specific microorganisms and metabolites that play a therapeutic role in FMT, and the prediction and evaluation factors for FMT efficacy remain largely unknown. Paramsothy et al. reported that specific bacteria and metabolites are related to the response to FMT in the treatment of UC. They found that those UC patients who achieved remission after FMT had enrichment of *Eubacterium hallii* and *Roseburia inulivorans* compared with patients who did not [38]. This suggests that the therapeutic effect of FMT may be related to the enrichment of certain bacteria. Besides, the treatment method is a nonnegligible issue. A collaborative analysis of 14 studies included 305 CDI patients treated with FMT, with 208 patients in the lower gastrointestinal route (LGI) and 97 patients in the upper gastrointestinal route (UGI) found a higher clinical failure in the UGI compared to LGI patients (17.9% vs. 8.5%). Moreover, a 3-fold increase in the hazard of clinical failure for UGI than LGI patients [39]. In our study, LFMT mice manifested reduced disease activity, alleviative intestinal mucosal barrier disruption (Fig. 3D and E), and recovery of bacterial diversity than UFMT mice (Fig. 4D and E). We observed an increased abundance of *Bacteroidota* and *Verrucomicrobiota* in UFMT and LFMT mice (Fig. 4F). We also observed a decreased abundance of *Firmicutes* and *Proteobacteria* in UFMT and LFMT mice (Fig. 4F). The alterations were more apparent in LFMT mice. In addition, the enriched bacterium in LFMT mice mainly includes symbiotic bacteria such as *Bacteroidota*, *Lachnospiraceae*, and *Prevotellaceae* (Fig. 4G), which suggests that these commensal bacteria may exert a therapeutical effect in the FMT treatment.

FMT can not only treat UC by restoring the diversity of the microbiota but also may treat colitis by eliminating pathogenic bacteria and pathogenic factors in the gut. Hourigan et al. found that FMT gives sustained *C. difficile* eradication in children with and without IBD. Besides, the *cdtB* virulence gene of *C. difficile* was undetectable in the patient’s feces at 3 and 6 months after FMT [23]. Julia et al. detected *Bacteroides fragilis*, *F. nucleatum*, *Escherichia coli*, and their virulence genes (*bft*, *fadA*, *pks*) pre-FMT and post-FMT in CDI patients, found that the levels of these oncobacterium and virulence genes were reduced or undetectable post-FMT [24]. In our experiment, we found *Lactobacillus*, *Allobaculum*, and *Bacteroidales* had a negative correlation with proinflammatory cytokines (Fig. 5A). Genera like *Romboutsia*, *Escherichia Shigella*, *Enterococcus*, and *Clostridium* were found positively correlated with proinflammatory cytokines (Fig. 5A). In addition, the levels of *F. nucleatum* (shown as *nusG* gene) and *fadA* gradually decreased with the increased times of FMT, and the decline curve of LFMT was more obvious than UFMT (Fig. 5C and D). Meanwhile, the levels of *F. nucleatum* and *fadA* were negatively correlated with the frequency of LFMT (Fig. 5E and F). Those results suggested that FMT contributes to diseases by reducing pathogenic bacteria and virulence factors.

## Conclusions

Generally, our results revealed that oral pathogens may affect the gut microbiota under certain circumstances and participate in the pathogenesis of UC. Meanwhile, as a novel therapeutic method, FMT has shown promising efficacy and safety in the treatment of UC. Our results also suggested that the therapeutic effect may be achieved by eliminating intestinal *F. nucleatum* and virulence factor and restoring intestinal microbiota diversity. However, the mechanisms that underlie the FMT in eliminating *F. nucleatum* and virulence factor need further explored. Besides, bacterial metabolites analysis was not incorporated into the experiment. Despite the limitations, this study provides a novel perspective to understand the “oral-gut-microbiome axis” and direction for predicting the therapeutic effect of FMT by using specific oral-associated bacteria as biomarkers.

## Data Availability

The raw data of 16S rRNA sequencing that support the findings in this study are available in the Science Data Bank at 10.57760/sciencedb.16482.
